# Morphine mediated neutrophil infiltration in intestinal tissue play essential role in histological damage and microbial dysbiosis

**DOI:** 10.1080/19490976.2022.2143225

**Published:** 2022-11-21

**Authors:** Richa Jalodia, Udhghatri Kolli, Regina Gonzalez Braniff, Junyi Tao, Yaa Fosuah Abu, Irina Chupikova, Shamsudheen Moidunny, Sundaram Ramakrishnan, Sabita Roy

**Affiliations:** a Department of Surgery, University of Miami Miller School of Medicine, Miami, FL, USA; b Department of Microbiology and Immunology, University of Miami Miller School of Medicine, Miami, FL, USA

**Keywords:** Opioids, inflammation, lamina propria, microbial dysbiosis, neutrophils, tight junction, lactobacillus, enterococcus

## Abstract

The gut microbial ecosystem exhibits a complex bidirectional communication with the host and is one of the key contributing factors in determining mucosal immune homeostasis or an inflammatory state. Opioid use has been established to induce gut microbial dysbiosis consistent with increased intestinal tissue inflammation. In this study, we investigated the role of infiltrated immune cells in morphine-induced intestinal tissue damage and gut microbial dysbiosis in mice. Results reveal a significant increase in chemokine expression in intestinal tissues followed by increased neutrophil infiltration post morphine treatment which is direct consequence of a dysbiotic microbiome since the effect is attenuated in antibiotics treated animals and in germ-free mice. Neutrophil neutralization using anti-Ly6G monoclonal antibody showed a significant decrease in tissue damage and an increase in tight junction protein organization. 16S rRNA sequencing on intestinal samples highlighted the role of infiltrated neutrophils in modulating microbial community structure by providing a growth benefit for pathogenic bacteria, such as *Enterococcus*, and simultaneously causing a significant depletion of commensal bacteria, such as *Lactobacillus*. Taken together, we provide the first direct evidence that neutrophil infiltration contributes to morphine-induced intestinal tissue damage and gut microbial dysbiosis. Our findings implicate that inhibition of neutrophil infiltration may provide therapeutic benefits against gastrointestinal dysfunctions associated with opioid use.

## Introduction

The opioid epidemic has its roots in the over prescription of opioids in the late 1990s, which led to countrywide misuse of both prescription as well as non-prescription opioids in the USA. Since the beginning, three distinct opioid waves have caused severe rise in opioid overdose deaths, with the first wave involving prescription opioids such as morphine and oxycodone, second wave involving heroin and the third wave which began in 2013 involving synthetic opioids particularly fentanyl which is 50–100 times more potent than morphine. Illicitly manufactured fentanyl is present in the street drugs such as heroin, cocaine, and methamphetamine, making them very potent, causing rapid respiratory depression followed by cardiac arrest, and has been responsible for the recent increase in drug overdose death. Even though opioids such as morphine remain the drug of choice for management of severe to moderate pain, their use is also associated with several comorbidities, particularly in the gastrointestinal (GI) tract, including constipation, nausea, bloating, and abdominal pain.^1,2^ The mammalian gut harbors a complex microbial ecosystem comprising bacteria, archaea, viruses, bacteriophages, and fungi, which together play a key role in host fitness, including synthesis of essential vitamins, toxin removal, defense against invading pathogens, intestinal barrier promotion as well as regulation of mucosal immune response.^3^ External factors such as diet, drug use, and lifestyle play important role in shaping microbial composition.^4–7^ In particular, we and others have shown that opioid-induced alterations in the gut microbiome compromise intestinal homeostasis, immunity, and predispose to infection.^8–10^ However, how opioids may regulate the mucosal immune system to impact microbial dysbiosis has not yet been established.

Multiple reports have shown that the host can affect gut microbial composition through its mucosal immune system.^4,11^ The innate and adaptive arm of the mucosal immune system interacts with gut commensal bacteria; by exhibiting tolerance toward commensals and proinflammatory responses toward pathogens, intestinal homeostasis is maintained.^12,13^ Resident gut microbiome also profoundly shapes intestinal mucosal immunity, as is apparent from studies showing a lack of organized lymphoid structures in the intestines of germ-free mice.^14,15^ Interplay between intestinal microbiome and immune system plays a critical role in determining healthy vs diseased conditions. Additionally, intestinal microbial dysbiosis has been shown to activate the immune system and promote a pro-inflammatory environment at the intestinal mucosal surface.^14,16^ While opioid modulation of immune cells such as neutrophils has been described systemically,^17^ how opioids affect the neutrophils within the GI tract has not yet been established. Neutrophils, in particular, are essential for the maintenance of intestinal homeostasis and response to pathogenic invasion through secretion of reactive oxygen species (ROS) and other toxic molecules such as myeloperoxidase (MPO), cathepsins, and defensins.^18^ Additionally, the importance of intestinal microbiota to neutrophil production and regulation of their recruitment and function have been well established.^19^ In turn, excessive tissue infiltration and activation of neutrophils under pathological conditions such as inflammatory bowel disease (IBD) are known to cause mucosal tissue damage.^16,20^ Therefore, neutrophils can be considered a double-edged sword in both protecting against pathogens and mediating tissue damage; a critical balance is essential to ensure safety from infiltrating pathogens and damage to tissue. How morphine may affect this balance in the GI tract is unknown.

Inflammatory environment which causes increased aerobic conditions, epithelial cell debris to feed on, and optimum mucus thickness may be beneficial for the proliferation of certain pathogenic bacterial species^21,22^ and has been associated with gut dysbiosis. Intestinal inflammation in case of chemically as well as genetically induced colitis has been reported to cause altered gut microbial composition.^23,24^ While the effects of morphine on peripheral immune cells have been studied at length, there have been no reported studies investigating the effect of morphine in modulating mucosal immune changes and how these changes contribute toward microbial dysbiosis. In the present study, we investigated the role of morphine mediated mucosal immune changes in tissue damage and gut microbial dysbiosis. Our results clearly establish that morphine treatment mediates changes in the gut microbiome that leads to inflammatory immune cell influx in intestinal tissue, which promotes intestinal tissue damage and intestinal bacterial dysbiosis.

## Results

### Morphine treatment compromises intestinal epithelial barrier function in mice

**Figure 1.**
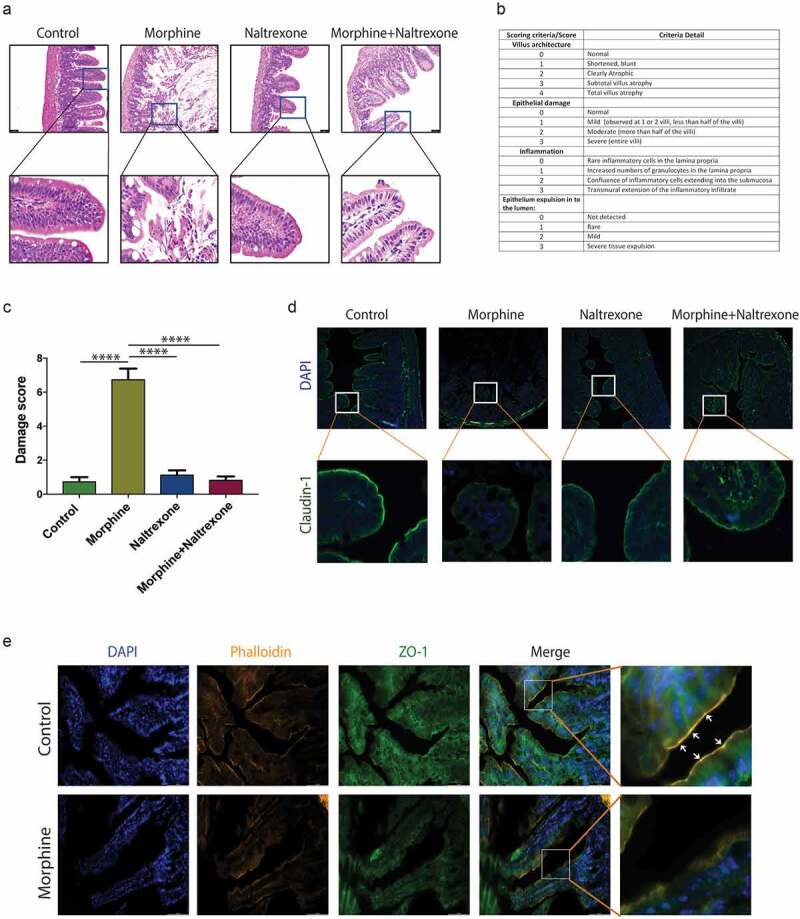
Morphine treatment compromises intestinal epithelial barrier function in mice. (a) Representative H&E stained small intestinal sections from control, morphine, naltrexone and naltrexone + morphine treated mice. (Scale bar: 50 μm). (b) Scoring guide for histopathological damage scoring from H&E stained intestinal sections. (c) Graph showing histopathological damage score in intestinal sections in different mice groups. (d) Representative image of Claudin-1 (green) organization in distal small intestine of control, morphine, naltrexone and naltrexone + morphine treated mice. (Scale bar: 50 μm). (e) Representative image for ZO-1 and F-actin staining from frozen ileum sections in control and morphine mice groups. White arrow indicates co-localization of ZO-1 with F-actin in control mice. Data represented as bar plots with Standard error of mean (SEM). Data were analyzed by one-way ANOVA with post-hoc Tukey’s test. ****P ≤ 0.0001.

We have previously shown that morphine treatment for 72 hours in mice causes bacterial translocation as a result of mucosal barrier compromise.^8^ In this study, 25 mg morphine slow-release pellet was implanted subcutaneously. Previous publication from our lab has confirmed the serum concentration of morphine (~100 nM-1 μM morphine over 7 days) in animals implanted with 25 mg slow-release pellet^8^ which is equivalent to human levels seen with slow-release prescription opioids either taken orally or opioids injected subcutaneously (range 50 nM-1 μM). Intestinal tissues were collected 24 hours later for histopathological analysis. Terminal ileal tissue sections stained with hematoxylin and eosin (H&E) were scored using established criteria (Figure 1b). In contrast to control mice, morphine treatment causes severe epithelial injury in intestinal villus in both male and female mice (Figure 1a, Supplementary figure 1A). Morphine treatment also led to significant inflammatory cell influx into intestinal tissue as well as apical villus expulsion into lumen, resulting in significant tissue damage in morphine treated mice compared to other treatment groups (Figure 1c). To investigate the role of μ-opioid receptor (MOR), we treated mice with the opioid antagonist, naltrexone. Our results demonstrate that naltrexone treatment antagonizes morphine-induced disruption of the intestinal barrier (Figure 1a, 1c). We also studied tight junction organization of intestinal epithelium by staining for tight junction proteins Claudin-1 and Zona occludens-1 (ZO-1). In control mice, Claudin-1 staining is continuous and localized to the apical side of the intestinal epithelial membrane (Figure 1d, Supplementary figure 1B). Similarly, ZO-1, the paracellular tight junction protein also localized with F-actin as marked by phalloidin staining in control mice (Figure 1e). In contrast, morphine treatment led to disrupted Claudin-1 and ZO-1 organization, suggesting impaired recruitment of tight junction proteins to the membrane (Figure 1d, 1e, Supplementary figure 1B). Compared to control mice, in naltrexone alone and naltrexone + morphine treated mice, no change was observed in Claudin-1 organization indicating the role of the MOR (Figure 1d).

### Morphine treatment increases neutrophil recruitment to intestinal tissue by regulating tissue chemokine expression

**Figure 2.**
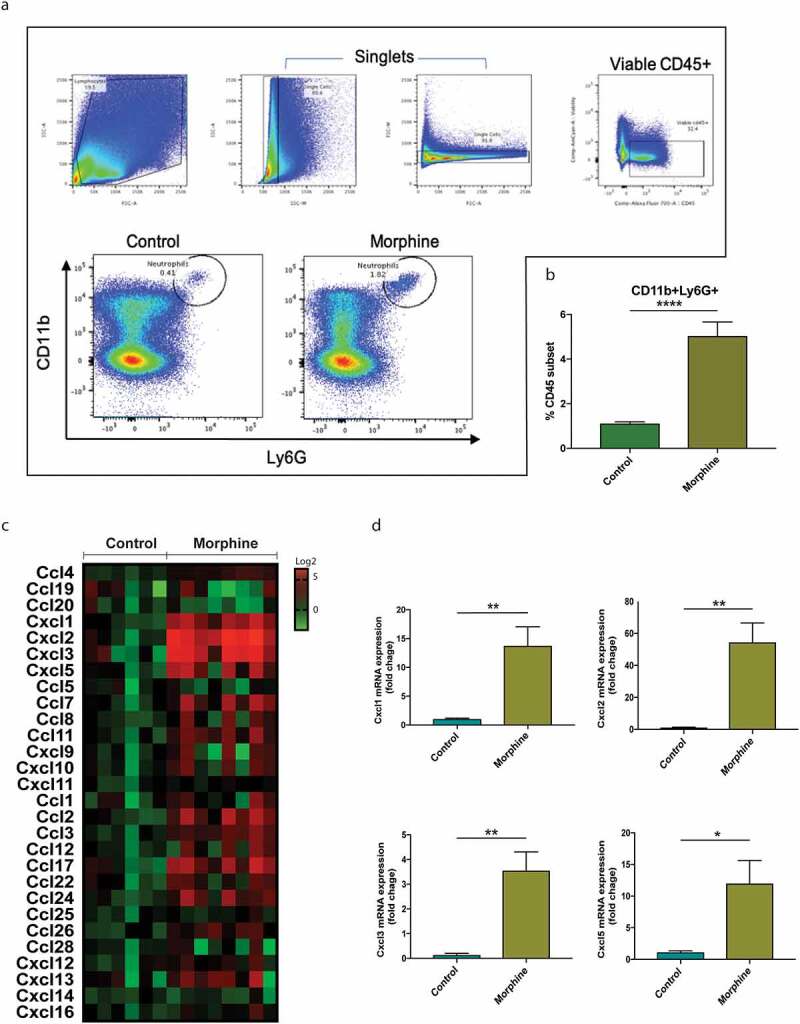
Morphine treatment increases neutrophil recruitment to intestinal tissue by regulating tissue chemokine expression. (a) Gating strategy for neutrophils isolated from lamina propria from mouse small intestine of control and morphine treated mice group with representative dot plot graphs for control and morphine group. (b) Graph showing CD11b+ Ly6G+ neutrophil cells, as a percentage of viable CD45 cell subset. (c) Heat map of chemokine levels in distal small intestinal homogenate from control and morphine group as determined by qPCR array. (d) Relevant chemokines differentially produced in distal small intestine in control and morphine group. Data represented as bar plots with SEM. (b,d)Data were analyzed by student’s t-test (*n* = 8–10). *P ≤ 0.05; **P ≤ 0.01; ****P ≤ 0.0001.

In intestinal tissue, chemokine and cytokine secretion by immune as well as intestinal epithelial cells (IECs) dictate local tissue inflammatory status and regulate immune cell homeostasis. To investigate the effect of morphine on intestinal immune homeostasis, immune cell profiling was done on cells circulating in intestinal lamina propria (LP) of morphine and control mice. The population of neutrophils identified by CD11b and Ly6G surface markers was found to be significantly high in morphine-treated mice independent of sex (Figure 2a, 2b, Supplementary figure 1C, 1D). Along with the neutrophil population, the monocyte cell population defined by Ly6C was also found to be significantly higher in morphine treated mice (Supplementary figure 2A, 2B). However, we did not find any significant changes in the macrophage population in the LP of intestinal tissue 24 h post morphine treatment (Supplementary figure 2C, 2D). In accordance with the immune cell changes, several chemokines involved in immune cell tissue infiltration were found to be dysregulated with morphine treatment (Figure 2c). Interestingly, the highest expressed chemokines were Cxcl1, Cxcl2, Cxcl3 and Cxcl5 (Figure 2d, Supplementary figure 1E), whose expression has been associated with neutrophil recruitment.^25^ Tissue chemokine expression at different time points (12 h, 72 h and 7 days) of morphine treatment revealed that high expression of neutrophil recruitment chemokine is an early response against tissue damage and invading pathogen (Supplementary figure 2E). Several other chemokines, including Ccl17 and Ccl22, which are involved in macrophage recruitment, were also found to be significantly upregulated post morphine treatment (Figure 2c).

### Gut microbiome regulates morphine-induced neutrophil infiltration and changes in chemokine expression

**Figure 3.**
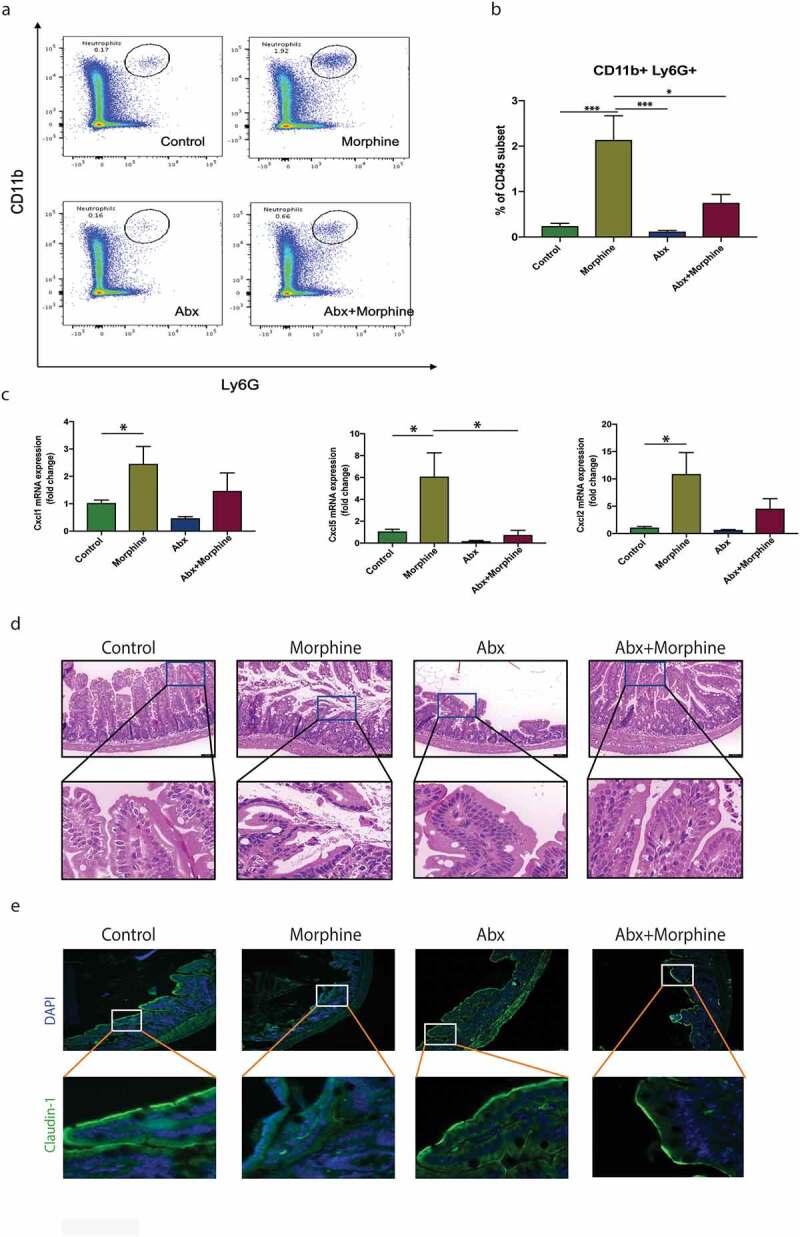
Gut microbiome regulates morphine-induced neutrophil infiltration and changes in chemokine expression. (a) Representative dot plot graphs showing flow cytometry for neutrophil population in different treatment groups. (b) Graph showing CD11b+ Ly6G+ neutrophil cells, as a percentage of viable CD45 cell subset in different treatment groups. (c) Relevant chemokines differentially produced in distal small intestine in different treatment groups. (d) Representative H&E stained small intestinal sections from control, morphine, Abx and Abx+morphine treated mice groups. (Scale bar: 50 μm). (e) Representative image of Claudin-1 (green) organization in distal small intestine of control, morphine, Abx and Abx+morphine treated mice. (Scale bar: 50 μm). Data represented as bar plots with SEM. Data were analyzed by one-way ANOVA with post-hoc Tukey’s test. *P ≤ 0.05; ***P ≤ 0.001.

We have previously shown that morphine treatment leads to gut microbial dysbiosis which causes systemic and local intestinal tissue inflammation.^26^ Additionally, others have shown that under infection and inflammatory conditions, mucosal immune cells respond to changes in the gut microbiome. To verify the role of morphine-induced microbial dysbiosis in modulating neutrophil changes in the intestinal mucosa, we used antibiotics-treated and germ-free (GF) mice models. Pan antibiotics + antifungal (Abx) treatment was given to specific pathogen free (SPF) mice for 8 days prior to morphine treatment (Supplementary figure 3A). Abx and SPF mice were implanted with a morphine or placebo pellet, and intestinal tissue was harvested 24 h later for analysis of tissue pathology and local immune cell profiling. The tissue neutrophil count, as measured by flow cytometry, was significantly reduced in mice pre-treated with Abx compared to morphine-treated mice (Figure 3a, 3b), highlighting the role of a dysbiotic microbiome in tissue neutrophil infiltration. The upregulated expression of neutrophil recruitment chemokines observed after morphine treatment was also abolished in mice pretreated with Abx (Figure 3c). Terminal ileal tissue was fixed and examined for histopathological changes. Consistent with our previously published report,^27^ Abx treatment abrogated any morphological damage caused by morphine treatment to intestinal tissue (Figure 3d) and rescued tight junction protein disruption as confirmed by Claudin-1 staining (Figure 3e). As a further proof of concept, in GF mice, morphine treatment did not cause any significant changes in tissue neutrophil count (Supplementary figure 3B, 3C). Additionally, no differences were observed in neutrophil recruitment chemokine expression in these mice upon morphine treatment (Supplementary figure 3D). Furthermore, morphine-mediated histopathological changes were also not observed in GF mice (Supplementary figure 3E).

### Morphine-induced tissue neutrophil infiltration causes tissue damage and disrupts tight junction organization

**Figure 4.**
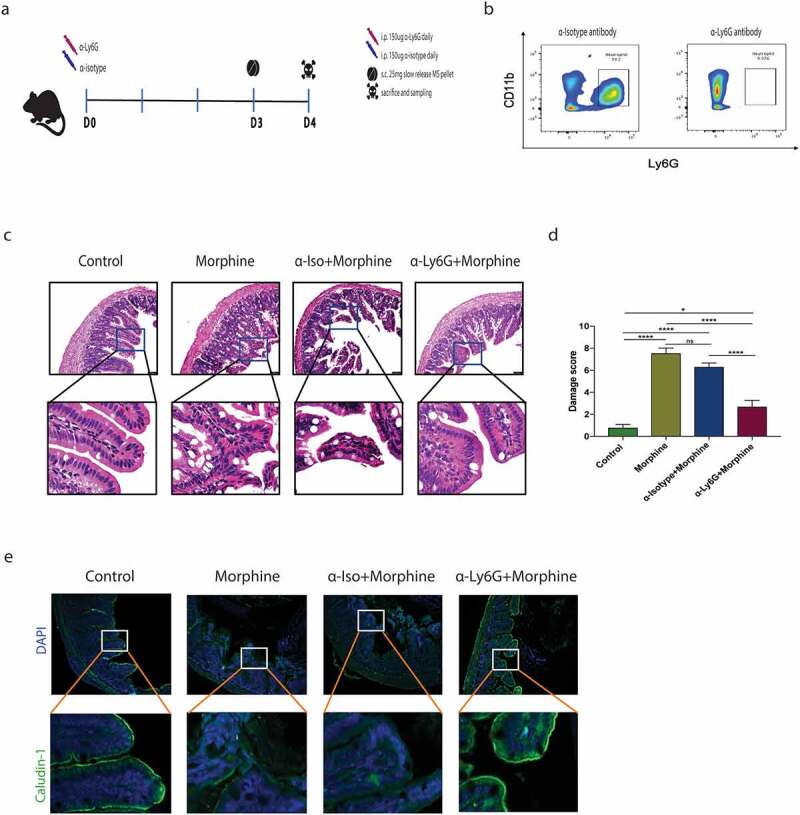
Morphine-induced tissue neutrophil infiltrates cause tissue disruption and disrupt tight junction organization. (a) Experimental scheme for neutrophil depletion in C57 mice with anti-Ly6G or anti-isotype antibody treatment for 3 days prior to morphine treatment (*n* = 5). (b) Flow cytometry analysis of CD45+ CD11b+Ly6G+ neutrophils in blood of mice treated with either anti-isotype or anti-Ly6G antibody. (c) Representative H&E stained small intestinal sections from control, morphine, anti-Isotype+morphine, and anti-Ly6G+morphine treated mice groups. (Scale bar: 50 μm). (d) Graph showing histopathological damage score in intestinal sections in different mice groups. (e) Representative image of Claudin-1 (green) organization in distal small intestine of control, morphine, anti-Isotype+morphine, and anti-Ly6G+morphine treated mice groups. (Scale bar: 50 μm). Data represented as bar plots with SEM. Data were analyzed by one-way ANOVA with post-hoc Tukey’s test. *P ≤ 0.05; ****P ≤ 0.0001.

To determine the role of neutrophils on the intestinal barrier function, neutrophils were depleted with a specific monoclonal antibody (mAb) against Ly6G prior to morphine treatment (Figure 4a). Flow cytometry analysis was done 3 days later to check for the efficacy of anti-Ly6G treatment. The number of neutrophils remained significantly lower in mice treated with anti-Ly6G mAb as compared to anti-isotype mAb treated mice (Figure 4b). H&E-stained terminal ileal tissue sections revealed that the depletion of neutrophils significantly attenuated morphine-induced histopathological tissue damage (Figure 4c, 4d). In line, damage to apical villi observed after morphine treatment was further abolished in absence of tissue neutrophil infiltrates. Consistently, Claudin-1 staining on fixed tissue sections further showed significant rescue in tight junction protein organization after neutrophil depletion (Figure 4e).

### Morphine-induced tissue neutrophil infiltrates contribute to gut microbial dysbiosis

**Figure 5.**
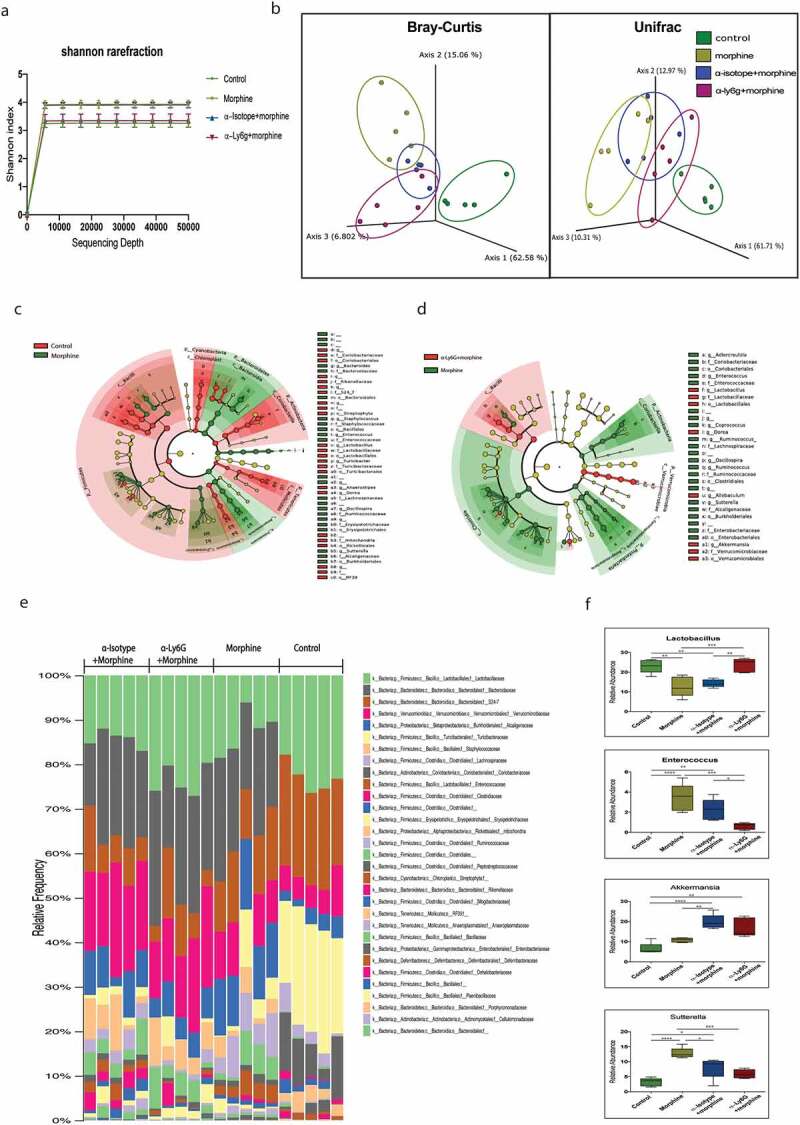
Morphine-induced tissue neutrophil infiltrates mediates gut microbial dysbiosis. 16S rRNA sequencing was performed from DNA sample extracted from small intestinal luminal sample from treated or untreated mice groups (*n* = 5) (a) Alpha diversity analysis using Shannon index shows increased alpha diversity in morphine and anti-isotype + morphine group compared to control and anti-Ly6G+morphine group. (b) Principal coordinates analysis (PCoA) of control samples shows distinct clustering of control group compared to morphine group using Unifrac metric (p = 0.008) and Bray-Curtis (p = 0.014). Comparison between OUT level showed distinct clustering between anti-Ly6G+morphine and morphine group using Unifrac (p = 0.041) and Bray-Curtis (p =0 .011) . Cladogram plotted from LEfSe analysis showing taxonomic comparison between (c) morphine and control treated mice, and (d) morphine and anti-Ly6G+morphine treated mice. (c,d) Morphine enriched taxa are green and control/anti-Ly6G+morphine enriched taxa are red. (e) Microbial composition in different treatment groups at the family level. (f) Tukey’s box plots showing relative abundance of bacterial genus significantly changing among different treatment groups. Data represented as bar plots with SEM. Data were analyzed by one-way ANOVA with post-hoc Tukey’s test. (*n* = 5). *P ≤ 0.05; **P ≤ 0.01; ***P ≤ 0.001; ****P ≤ 0.0001.

Neutrophils are considered to be the first responders in the immune system, providing immediate defense against damage-associated molecular patterns (DAMP) and pathogen-associated molecular pattern (PAMP) signals. However, paradoxically, through their secretory factors including ROS, neutrophil extracellular traps (NETs), and proteinases, neutrophils also can contribute to host tissue damage. To determine whether the infiltrated neutrophils contribute to tissue damage and microbial dysbiosis, mice were treated with anti-Ly6G mAb for 3 days prior to implanting morphine or placebo pellet. 24 h after morphine treatment, small intestinal fecal content was harvested. Extracted fecal DNA was subjected to 16S rRNA sequencing. Our data revealed that morphine and anti-isotype + morphine (IM) treated mice exhibit a significant increase in alpha diversity as measured by the Shannon index compared to control and anti-Ly6G+morphine (LM) treated mice group (Figure 5a, Supplementary figure 4A). Beta diversity analysis revealed that morphine treatment results in significantly distinct clustering of bacterial communities compared to control mice using weighted Unifrac distance (*P* = 0.008) as well as Bray-Curtis (*P* = 0.014) (Figure 5b). Unifrac analysis also reveals high similarity in clustering of the IM group to morphine-treated mice (*P* = 0.058); in contrast, LM treated mice showed a significant shift in microbial composition from that of morphine-treated group in the Unifrac analysis (*P* = 0.041) (Figure 5b). A reduced Firmicutes to Bacteroidetes ratio was observed after morphine treatment compared to control, with no significant changes between morphine, LM, and IM groups (Supplementary figure 4B). Further analysis revealed that morphine treatment resulted in a significant increase in the pathogenic genus *Staphylococcus, Enterococcus,* and Bacteroides (Figure 5c). Comparison of taxonomic groups between morphine vs control, and morphine vs LM mice further showed consistent changes in several bacterial genus for e.g. *Enterococcus, Lactobacillus,* and *Dorea* after morphine treatment (Figure 5c, 5d). No significant changes were observed in *Enterococcus, Lactobacillus,* and *Dorea* in morphine vs IM group microbiome (Supplementary figure 4D). Stack bar graphs show the relative abundance of several bacterial taxa at the family level per different treatment group (Figure 5e). A group-wise comparison revealed that the decreased abundance of the *Lactobacillus* genus and increased abundance of the *Enterococcus* genus observed after morphine treatment is also seen in IM-treated mice but not in LM-treated mice (figure 5f). Enrichment of pathogenic firmicutes as represented by the Enterococcus/Lactobacillus ratio is observed in morphine-treated mice compared to control and LM mice groups (Supplementary figure 4C). Interestingly, both LM and IM-treated mice showed an increased abundance of the *Akkermansia* genus and a decreased abundance of the *Sutterella* genus compared to the morphine-treated group (figure 5f). Our data imply the potential role of neutrophil infiltrates in modulating gut microbial composition.

## Discussion

While the analgesic and sedative properties of morphine are well established, morphine use is still associated with several co-morbidities, particularly in the GI tract. Previous studies from our lab and others have demonstrated that both short- and long-term exposure to morphine alters the gut microbial composition, with an expansion of pathogenic bacterial communities,^8,26,28–30^ and parallelly leads to immune cell infiltration in the intestinal tissue.^8,31^ In addition to prescription opioids, long-acting μ opioid receptor agonists used for medication-assisted treatment for opioid use disorder (OUD), such as buprenorphine and methadone have also been shown to cause gut microbial dysbiosis and altered metabolic profiles in recent human studies.^32,33^ Altered immune homeostasis, in response to a dysbiotic microbiome, can trigger immunopathology. As such, understanding how dysbiosis mediates intestinal immune cell recruitment after morphine treatment is of great interest and clinical relevance. However, a detailed analysis of immune cell changes in intestinal LP following morphine treatment has yet to be conducted due to procedural difficulties, including cell’s susceptibility to reduced viability upon preservation, which mandates quick assessment after fresh isolation from tissue. Here, we provide mechanistic insight into the contribution of infiltrated neutrophils to intestinal pathophysiology and epithelial tight junction function after morphine treatment. In the current study, we used a short-term morphine treatment for 24 hours and analyzed mucosal immune changes in murine small intestine. We show that short-term morphine treatment not only caused significant damage to intestinal tissue with severe injury to the villus but also resulted in increased immune infiltration in male as well as female mice. In line, we also observed drastic disruption of tight junction organization in morphine-treated mice, consistent with other reports of bacterial translocation after morphine treatment.^31^ Indeed, recent studies have shown that intestinal microbial homeostasis is one of the key determinants of intestinal barrier functional integrity.^8,34^

IECs have been reported to provide chemokine signals for the initial wave of leukocyte recruitment.^25^ In our study, gene analysis data of intestinal tissue revealed several chemokines significantly altered by morphine treatment; we observed an increased expression of granulocyte recruitment chemokines Ccl8, Ccl11, Ccl24, and Ccl26, and increased transcription levels of Ccl17 and Ccl22, which are involved in tissue macrophage recruitment. However, no changes were observed in the tissue macrophage population as confirmed by flow cytometry. In our data, we also observed a significant increase in the Ly6C^hi^ monocyte population. This observation is consistent with previously published studies where intestinal inflammation is associated with increased monocyte infiltration in LP.^35,36^ These newly recruited monocytes have been shown to differentiate into Ly6C^lo^MHCII^+^ expressing mature macrophages in a process known as monocyte to macrophage waterfall^37^ and occur in 5–6 days with changes in genes involved in phagocytosis, complement pathways, and IL-10.^38^ Based on the published studies it seems highly likely that changes in the mucosal macrophage population will be observed at later time points after morphine treatment, and further work will be required to understand the time kinetics of mucosal macrophage population change after gut dysbiosis. Interestingly, the greatest fold change of chemokines analyzed were Cxcl1, Cxcl2, Cxcl3, and Cxcl5, all of which are associated with neutrophil recruitment. In accordance with these changes, we also observed a significant increase in the LP neutrophil population in intestinal tissue. Microbiome depletion using Abx prior to morphine treatment significantly decreased tissue expression of neutrophil recruitment chemokines as well as neutrophil counts in the LP compared to treatment with morphine alone. To further confirm the role of the microbiome in altering the tissue chemokine profile and neutrophil recruitment, we used a germ-free mice model. Similar to Abx-treated mice, in GF mice no changes in intestinal chemokine expression or neutrophil infiltration were observed following morphine treatment. Additionally, we also observed significantly less histopathological damage in GF as well as in the Abx + morphine mice, further highlighting the role of a dysbiotic microbiome in contributing to intestinal tissue damage and tight junction disruption. Together, our data clearly suggests the role of morphine-mediated dysbiosis in tissue inflammation and neutrophil recruitment.

Microbial dysbiosis and pathogenic microbial products have been linked to an inappropriate immune response in colitis-associated cancer^39^ as well as in IBD;^16^ both conditions have also been associated with an increase in tissue chemokine levels.^16,39^ Similarly, neutrophil infiltration into intestinal tissue, in response to dysbiotic microbiome and tissue injury, has also been observed in IBD, graft-versus-host disease, and necrotizing enterocolitis.^40–44^ Indeed, neutrophils play a crucial role in the response toward pathogenic bacteria. However, accumulated neutrophils can cause an increased release of cytotoxic products that can interact synergistically with microbial agents and promote local tissue damage.^45^ For instance, NETs released by recruited activated neutrophils have been shown to contribute to intestinal injury in abdominal sepsis patients,^46,47^ IBD patients^48^ as well as in colorectal cancer patients.^49^ In some cases, NETs have even been shown to assist pathogenic bacterial infection by assisting their attachment to the intestinal mucosa.^50^ In our studies, since neutrophils were the main population of immune infiltrates 24 h after morphine treatment, we used an anti-Ly6G antibody for depleting neutrophils to further understand their role in tissue injury and dysbiosis. Our data showed that anti-Ly6G treatment prior to morphine treatment significantly rescued intestinal histopathological damage, with a significantly lower damage score in this group, compared to both the morphine group and the anti-isotype + morphine group. An *in-vitro* report also suggested that trans migration of neutrophils can damage tight junctions in cultured epithelial cells,^51^ which was further reflected here in our Claudin-1 and ZO-1 staining data. Specifically, our data show that neutrophil depletion prior to morphine treatment rescued the decreased Claudin-1 expression and localization typically observed after morphine treatment. Taken together, our data strongly implicate the tissue-damaging role of infiltrated neutrophils in intestinal tissue post morphine-treatment.

Whether infiltrated immune cells influence microbial dysbiosis has not been explored previously; thus, we conducted 16S rRNA analysis on intestinal luminal samples in the presence or absence of infiltrated tissue neutrophils. We observed an increased richness in bacterial alpha diversity after morphine treatment as well as in the anti-isotype + morphine group compared to control and anti-Ly6G+morphine groups. Additionally, our data also showed distinct microbial community clustering after morphine treatment compared to control animals, in agreement with previous findings.^8,26,31^ Interestingly, anti-Ly6G and not anti-isotype pre-treatment before morphine pellet implantation was able to shift the bacteria community away from the morphine group. However, this was only a partial shift, as communities were still distinct and separable from the control group. At the phylum level, a significant decrease in Firmicutes/Bacteroidetes ratio was observed in the morphine group compared to the control group. No significant change was observed between morphine, IM, and LM groups. Firmicutes/Bacteroidetes ratio has been used as a marker for obesity, diabetes, and aging.^52,53^ However, contradictory results have been reported on the Firmicutes/Bacteroidetes ratio in opioid studies.^8,29^ Further analysis of the Firmicutes phylum revealed an overgrowth of the pathogenic genus *Enterococcus* and a significant depletion of the commensal genus *Lactobacillus* in the intestinal lumen of morphine-treated mice. Notably, treatment of mice with anti-Ly6G (and not the anti-isotype antibody group) prior to morphine treatment protected against these bacterial genus changes in mice. However, some taxa were affected by both IM and LM treatment, including *Akkermansia* and *Sutterella* at the genus level. Injecting mice with control or specific antibodies prior to morphine treatment may have contributed to these alterations.

*Enterococcus* is a well-studied pathogenic bacterial genus that is a known contributor toward intestinal inflammation, compromising epithelial barrier by damaging tight junction function.^54^ Opposite to that, bacterial species belonging to *Lactobacillus* genus strengthen the intestinal barrier and tight junction integrity.^34^ Previous research has shown varied neutrophil response to different infectious agents such as *E.* *coli, S.* *aureus*, and group A streptococcal cases which plays important role in the pathophysiology during infection.^55^ In our data, we observed a specific response of tissue-infiltrated neutrophils in contributing to the depletion of the commensal genus *Lactobacillus* and at the same time giving the growth benefit for the pathogenic genus *Enterococcus*. Therefore, the role of tissue-infiltrated neutrophils in causing gut microbial shift might be the underlying factor for tight junction damage observed after morphine treatment. Taken together, our data suggest that these immune changes initiated by altered microbial composition may further result in the selection of a dysbiotic bacterial community associated with the propagation of a disease phenotype.

Our study provides essential information regarding the bidirectional interkingdom communication between host and microbiota. Previous studies have also reported opioid-induced gut microbial dysbiosis in rodents and primates along with the observed intestinal barrier disruption.^8,31,56–58^ Comparing our data with the previously reported bacterial changes, we find a consistent decrease in Clostridiales and Ruminococcaceae as observed in chronic opioid users and non-human primates;^58,59^ decrease in *Lactobacillus* and an increase in *Enterococcus* and *Staphylococcus* as observed in rodent opioid studies.^9,56^ Other recent human studies also reported changes in Bifidobacterium and Prevotella taxa in chronic opioid users^57^ as well as in OUD patients taking buprenorphine or methadone,^32,33^ which were not observed in our study. Despite variability in opioid-induced dysbiotic community observed in different species, consistent depletion of bacterial communities associated with the metabolism of bile acids and short-chain fatty acid were observed.^8,32,33^ However, mechanistic work connecting dysbiosis and mucosal immune function is implausible in patients using prescription opioids, opioid abusers or OUD patients on buprenorphine or methadone. Our data can be extrapolated to provide mechanistic insight in those instances since microbial dysbiosis and inflammation are the common denominators. Therefore, our study can not only be helpful in understanding the role of bacterial communities in mediating mucosal immune changes but also help design future studies for in-depth exploration of microbiome-host interactions.

Additionally, recent research also points toward the role of tissue inflammation and gut dysbiosis in inducing tolerance to morphine.^27,33,60^ Tolerance to antinociceptive effects of opioids due to chronic use eventually leads to dependence and drug abuse. Data from our study strongly indicate that regulation of immune infiltration can help maintain gut microbial and immune homeostasis and thus can potentially delay the development of tolerance to opioids. Therefore, research efforts should be focused on exploring strategies such as reducing immune infiltration to reduce tissue inflammation and studying its effects on opioid tolerance and dependence.

A few limitations of the current study include a lack of (i) data for metabolic changes in different groups (ii) longitudinal kinetics of microbial and immune alteration which would have helped in the exploration of the cause-effect relationship between neutrophil tissue infiltration and microbial changes in the gut. However, our data underline the importance of future studies focusing on specific agents capable of blocking chemokine and chemokine receptor interactions that can restrict tissue neutrophil infiltration in patients who rely on opioid treatment.

## Material and methods

### Experimental animals

C57BL/6 male or female mice (stock no: 000664) were purchased from Jackson Laboratories (Bar Harbor, ME). All mice were 8-week-old pathogen-free male/ female. A maximum of 5 mice were housed per cage with food and tap water available ad libitum. Animal housing facilities were maintained in a 12-h light/dark cycle, with constant temperature (72 ± 1°F) and 50% humidity. All animal studies were approved by the Institutional Animal Care and Use Committee at the University of Miami. All procedures were conducted in accordance with the guidelines set forth by the National Institute of Health Guide for the Care and Use of Laboratory Animals.

### Animal treatment

The animals were lightly anesthetized with isoflurane (Pivetal®) and subcutaneously implanted with 25-mg slow-release morphine pellet or placebo pellet. The pellets were obtained from National Institute on Drug Abuse. All efforts were made to minimize suffering during and after surgery. To deplete gut microbiota, a pan-antibiotics + antifungal cocktail [vancomycin 32 (mg/kg), bacitracin (80 mg/kg), metronidazole (80 mg/kg), neomycin (320 mg/kg), Pimaricin (0.192 mg/kg)] was prepared every day in drinking water and an oral gavage was given to mice for 8 days prior to treatment with morphine or placebo pellet as described previously.^27^ For neutrophil depletion assays, 150 μg of anti-Ly6G (clone 1A8, # BP0075-1, Bio X Cell) and corresponding isotype control (clone 2A3, # BP0089, Bio X Cell) were injected intraperitoneally 3 days prior to morphine or placebo pellet implant with dose as described in the schematic in figure or legends.

### Immunofluorescence

Sections from the distal small intestine from each treatment group were fixed in 10% formalin and embedded in paraffin wax. A minimum of 10 sections from each of the 5 animals per group were analyzed by immunofluorescence microscopy. Representative images are shown. For immunostaining with Claudin-1 antibody, 8 μm tissue sections were deparaffinized with xylene and tissue was rehydrated through a series of graded alcohol and then processed for antigen retrieval using citrate antigen retrieval buffer (DAKO). Tissue sections were stained with polyclonal rabbit antibody against Claudin-1 (Invitrogen) in PBS with 1% bovine serum albumin (BSA) overnight at 4°C. For immunostaining with anti-ZO-1 antibody, intestinal tissue was frozen in TFM tissue freezing media, and later 5 μm tissue sections were used. Sections were fixed with 1% paraformaldehyde for 10 min at room temperature and incubated with anti-ZO-1 antibody (Invitrogen). After washing, sections were incubated with Alexa Fluor 488-conjugated goat anti-rabbit IgG secondary antibody (Invitrogen); for F-actin staining, sections were incubated with Alexa 555 conjugated phalloidin (Invitrogen) for 1 h at room temperature. Sections were mounted under coverslip using ProLong Gold antifade reagent with DAPI (Invitrogen). Stained sections were imaged using a fluorescence microscope (Leica Microsystems, Germany).

### Histology analysis

For examining the impact of morphine treatment on mouse small intestine, distal ileal tissue was harvested from mice after euthanizing. Tissue was fixed in 10% formalin and embedded in paraffin. 8 μm section of tissue was mounted on a clean glass slide and stained with hematoxylin and eosin (H&E) for histological evaluation. A blinded evaluation was done for each tissue section slide. For assessing the damage induced by intestinal inflammation, a histological score was given based on cumulative scores specified to various histological features as mentioned in Figure 1b. The combined histological score ranges from 0 (no damage) to 13 (extensive tissue damage and immune cell infiltration) as described in previous studies.^61–63^

### Real-Time PCR

Total RNA from terminal intestinal tissue section was extracted using TRIzol™ (Invitrogen). cDNA was synthesized using High-Capacity cDNA Reverse Transcription Kit (applied biosystems) as per the manufacturer’s protocol. Primers for chemokine analysis were purchased from Sigma. Quantitative real- Normalization was done using GAPDH and results were analyzed by relative quantity (ddct) method.

### Lamina propria cell isolation and flow cytometry staining

Immune cells were isolated from terminal small intestinal tissue sections using mouse Lamina Propria Dissociation kit (Miltenyi) according to the manufacturer’s instructions. Following isolation, immune cells were washed with PBS and resuspended in PBS+1%BSA, and stored at 4°C before proceeding with flow cytometry staining. For flow cytometry staining, ~1X10^6^ cells were placed in a 96-well plate and Fc receptor blocking as done using mouse Fc Block (BD biosciences) for 15 min at 4°C. Cells were then stained with an appropriate Ab mixture for surface staining at 4°C for 30 mins. Cells were washed after staining with staining buffer and fixed with fixation buffer (Biolegend). Cells were analyzed on BD LSR II (BD biosciences) and flow cytometry data was analyzed using FlowJo software (version 10.8.0; Tree star, Ashland, OR).

### 16S rRNA gene sequencing

Content of distal small intestine was collected under aseptic conditions after sacrificing mice. DNA isolation was done using DNeasy PowerSoil kit (Qiagen). Sequencing was performed at the University of Minnesota Genomic Center, MN, United States where 16S ribosomal DNA hypervariable regions V4 were amplified, and samples were sequenced by illumine MiSeq generating 300 base paired-end reads.

### Bioinformatics analysis

Demultiplexed sequence reads were clustered into amplicon sequence variants (ASVs) with the DADA2 package (version 1.21.0)^64^ implemented in R (version 4.0.3) and RStudio (version 1.1.463). The steps of the DADA2 pipeline include error-filtering, trimming, learning of error rates, denoising, merging of paired reads, and removal of chimeras. The ASV table generated by DADA2 was imported into the QIIME2 pipeline^65^ for diversity analyses and taxonomic assignment. Diversity analyses were performed by using the qiime diversity core-metrics-phylogenetic script with a sampling depth of 50,000. Taxonomic assignment of ASVs was done to the genus level using a naive Bayesian classifier^66^ implemented in QIIME2 with the Greengenes reference database (13_8 99%).^67^ LDA Effect Size (LEfSe)^68^ was generated by uploading the taxonomic assignment table to the galaxy app (https://huttenhower.sph.harvard.edu/galaxy/) to detect differentially abundant taxa across groups. The threshold on the logarithmic LDA score for discriminative features was set to 2.

### Statistical analysis

GraphPad Prism (GraphPad Software, Inc.) was used for experimental data analysis and plotting. Parametric data was compared using Student’s t-test. Quantitative data are expressed as means ± SEM of three experiments. Kruskal-Wallis test was used to detect if *α* diversity differed across treatments. Permutational multivariate analysis of variance (PERMANOVA) was used to detect if *β* diversity differed across treatments. Benjamini-Hochberg method was used for controlling false discovery rate (q-value). Student’s t-test was used to analyze the data differences between the two groups. One-way ANOVA followed by Tukey’s or Dunnett’s multiple comparison test was used to analyze the data with more than two groups. All data are presented as mean ± standard error. Statistically significant differences are represented as *P ≤ 0.05, **P ≤ 0.01, ***P ≤ 0.001, ****P ≤ 0.0001.

## Supplementary Material

Supplemental Material

## Data Availability

The authors confirm that the data supporting the findings of this study are available within the article and its supplementary materials.
