# Supplementary material for: Morphine mediated neutrophil infiltration in intestinal tissue play essential role in histological damage and microbial dysbiosis
Source: Gut Microbes. 2022 Nov 21;14(1):2143225. doi: 10.1080/19490976.2022.2143225 (PMC9683065; doi:10.1080/19490976.2022.2143225)
Supplement: Supplemental Material [file KGMI_A_2143225_SM4635.pdf]

Supplementary Figure 1

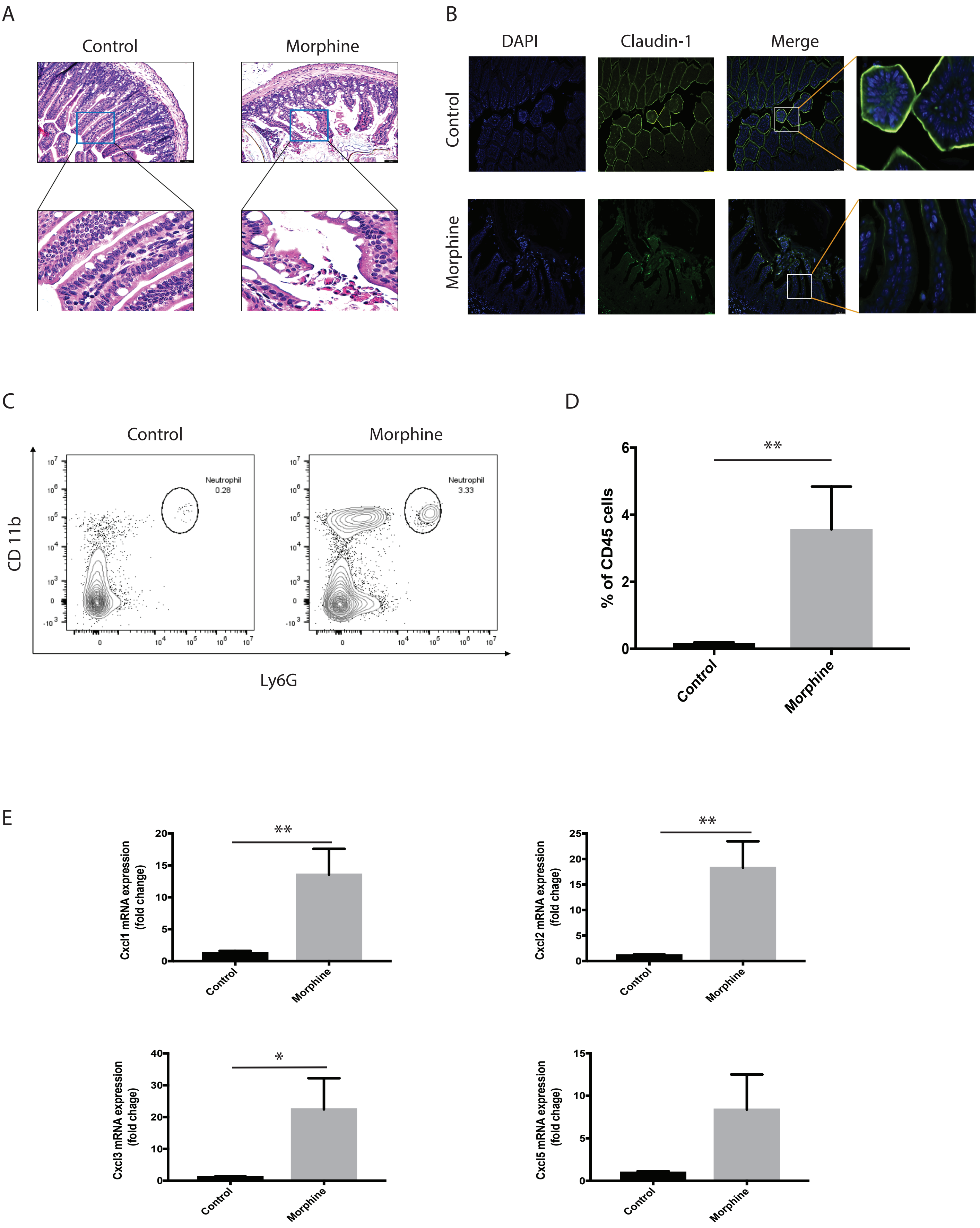

Supplementary Figure 2

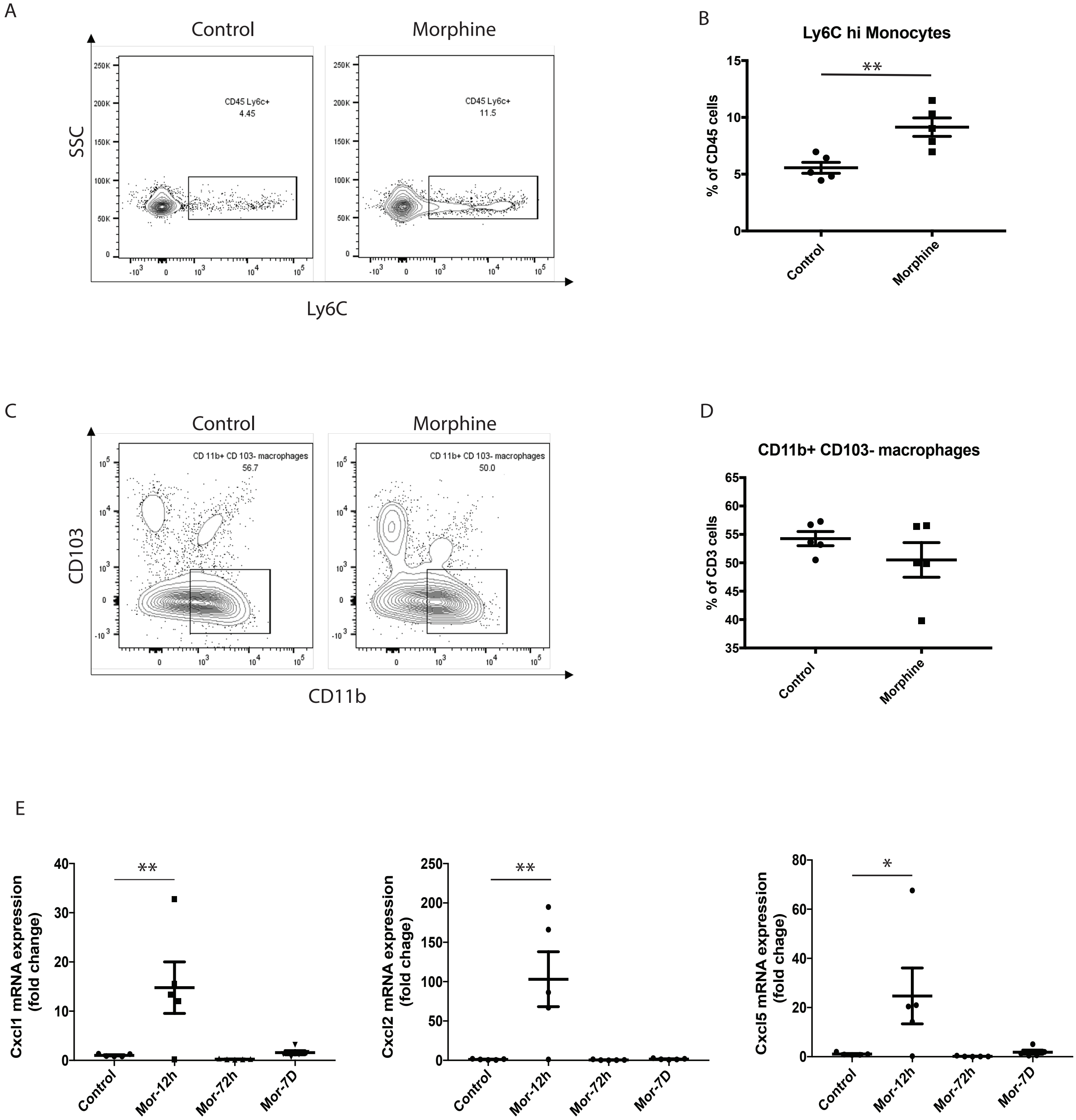

Supplementary Figure 3

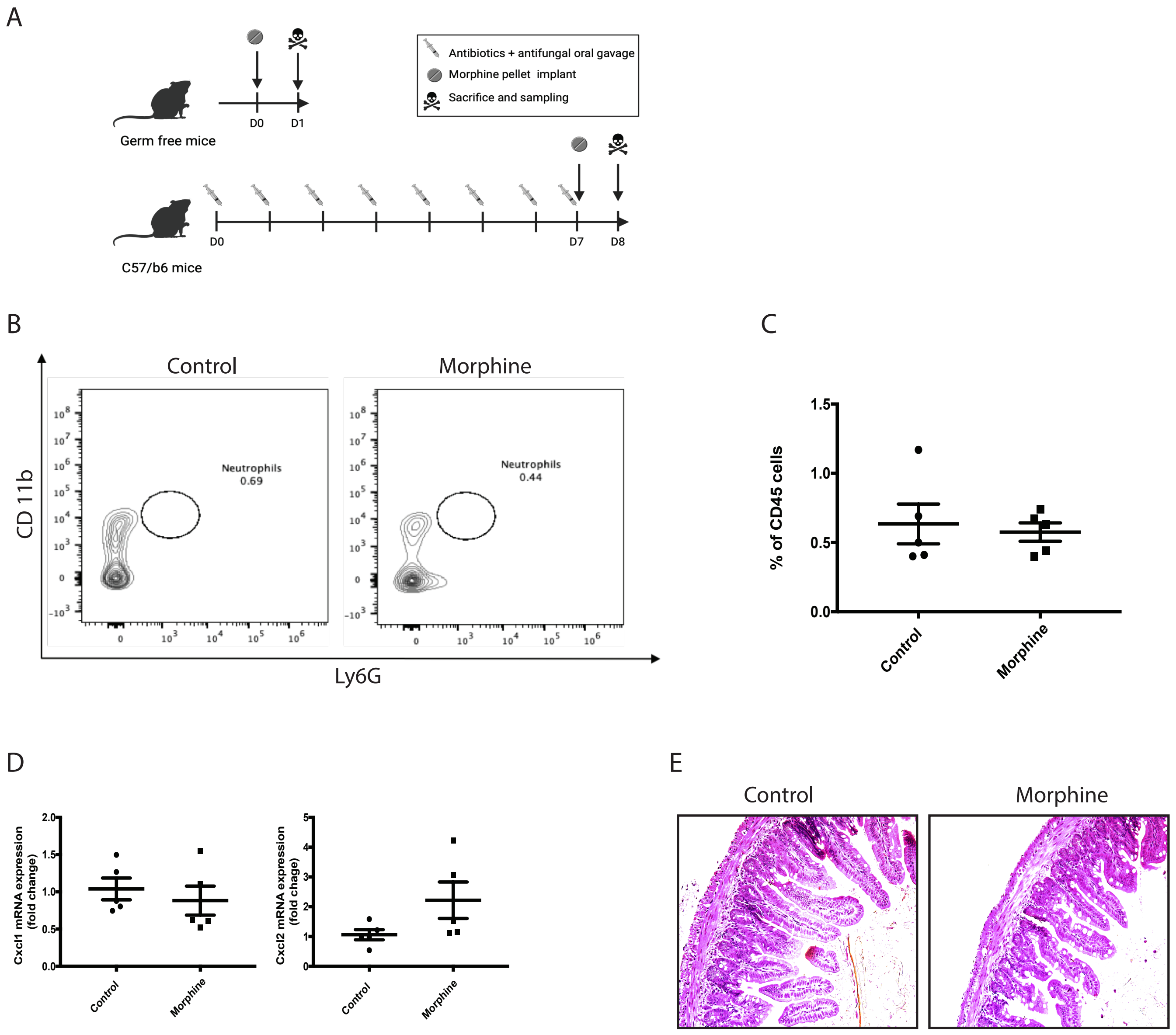

Supplementary Figure 4

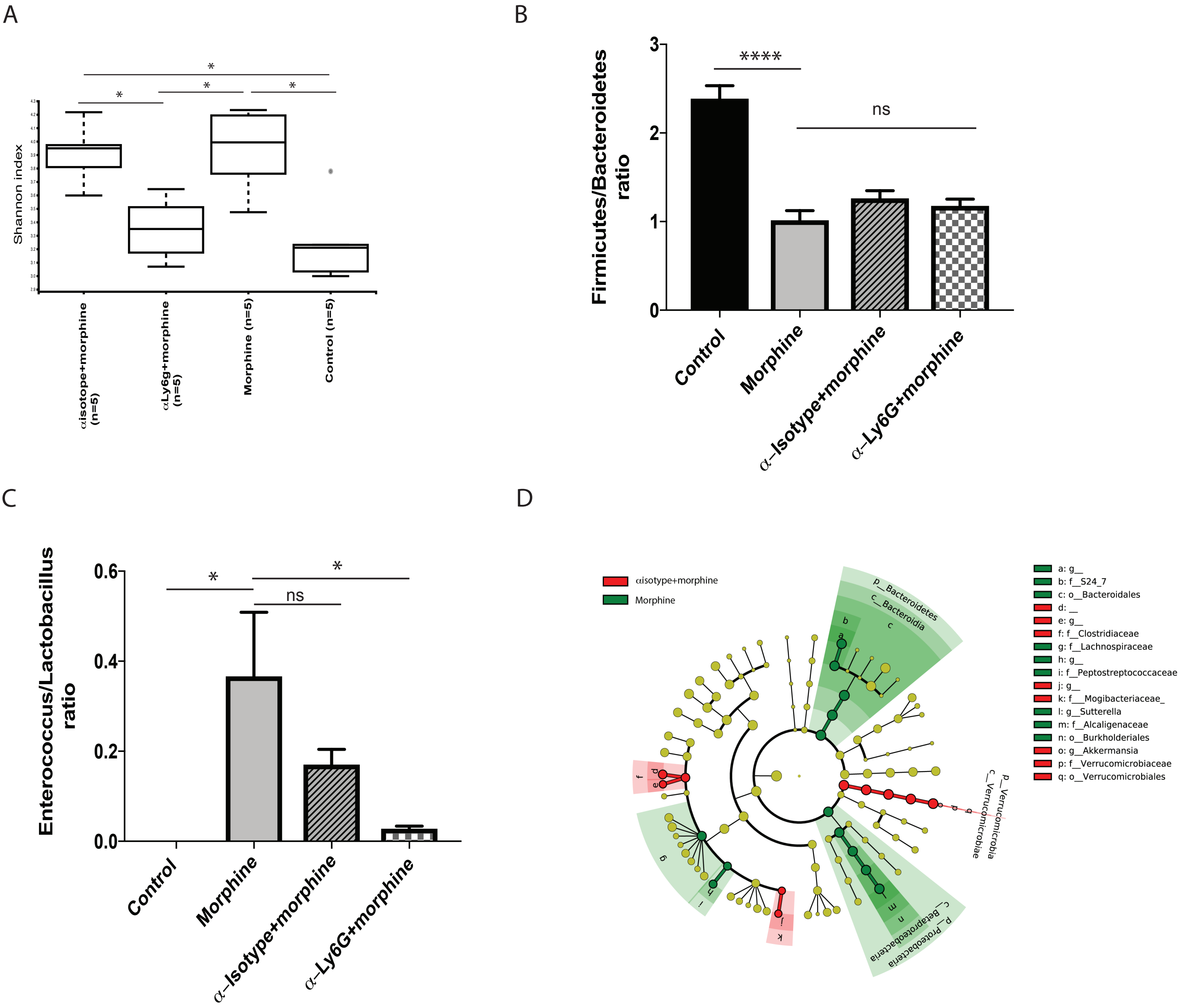

Supplementary Figure 1)

Morphine treatment cause histopathological damage, compromises intestinal epithelial barrier function, chemokine alteration and neutrophil infiltration in female mice. (A) Representative H&E stained small intestinal sections from control and morphine treated C57 female mice (Scale bar: 50 μm). (B) Representative image of Claudin-1 (green) organization in distal small intestine of control and morphine treated female mice. (Scale bar: 50 μm). (C) Representative dot plot graphs showing flow cytometry for neutrophil population in control and morphine treatment groups. (D) Graph showing CD11b+ Ly6G+ neutrophil cells, as a percentage of viable CD45 cell subset. (E) Real time PCR data showing expression of Cxcl1, Cxcl2, Cxcl3, and Cxcl5 chemokines differentially produced in distal small intestine in placebo and morphine treated female mice. Data represented as bar plots with Standard error of mean (SEM). Data were analyzed by student's t-test (*n*=8-10). $*P \leq 0.05$; $**P \leq 0.01$.

Supplementary Figure 2)

Morphine treatment alters chemokine expression in mouse small intestine and impact immune cell changes in intestinal tissue (A) Representative dot plot graphs showing flow cytometry analysis for monocyte population in placebo and morphine treated mice. (B) Graph showing percentage of Ly6C+ cells in different treatment groups. (C) Representative dot plot graphs showing flow cytometry for macrophage population in different treatment groups. (D) Graph showing percentage of macrophage cells in different treatment groups. (E) Real time PCR data showing expression of Cxcl1, Cxcl2 and Cxcl5 chemokines differentially produced in distal small intestine in placebo and morphine treated mice at different time points. Data were analyzed by student's t-test (B, D) and by one-way ANOVA with post-hoc Tukey's test (E) (*n*=5). $*P \leq 0.05$; $**P \leq 0.0$. Mean ± SEM.

Supplementary Figure 3)

Morphine treatment mediated histopathological and immune cell changes in intestinal tissue are not observed in germ-free mice. (A) Experimental scheme for C57/b6 mice treated with antibiotics and antifungal cocktail for 7 days prior to morphine treatment and germ-free mice treatment with slow release morphine pellet (*n*=5-8). (B) Graph showing percentage of CD11b+ Ly6G+ cells in germ-free mice ileal LP. (C) Graph showing percentage of CD11b+ Ly6G+ cells in different treatment groups in germ-free mice. (D) Graph showing Cxcl1 and Cxcl2 chemokines mRNA expression in distal small intestine of germ-free mice treated with placebo or morphine pellet. (E) Representative H&E stained small intestinal sections from germ-free mice treated with placebo or morphine pellet. (scale bar: 50 μm). Data were analyzed by student's t-test (*n*=5). Mean ± SEM.

Supplementary Figure 4)

Morphine-induced tissue neutrophil infiltrates mediates gut microbial dysbiosis. (A) Box plot of alpha diversity calculated by the Shannon index. (B) A significant decrease Firmicutes : Bacteroidetes ratio was observed in morphine, IM and LM groups compared to control microbiome. (C) Bar graph showing significant increase in Enterococcus : Lactobacillus genus ratio in morphine treated group compared to control and LM group. (D) Cladogram plotted from LEfSe analysis showing taxonomic comparison between morphine and IM treated mice groups. Morphine enriched taxa are green, IM enriched taxa are red. Data were analyzed by (A) Kruskal-Wallis test, (B) one-way ANOVA with post-hoc Tukey's test. *$P \leq 0.05$; ****$P \leq 0.0001$ (n=5). Mean ± SEM.
